# High e+/e− Ratio Dense Pair Creation with 10^21^W.cm^−2^ Laser Irradiating Solid Targets

**DOI:** 10.1038/srep13968

**Published:** 2015-09-14

**Authors:** E. Liang, T. Clarke, A. Henderson, W. Fu, W. Lo, D. Taylor, P. Chaguine, S. Zhou, Y. Hua, X. Cen, X. Wang, J. Kao, H. Hasson, G. Dyer, K. Serratto, N. Riley, M. Donovan, T. Ditmire

**Affiliations:** 1 Rice University, Houston, TX 77005, USA; 2 University of Texas at Austin, TX 78712, USA

## Abstract

We report results of new pair creation experiments using ~100 Joule pulses of the Texas Petawatt Laser to irradiate solid gold and platinum targets, with intensities up to ~1.9 × 10^21^ W.cm^−2^ and pulse durations as short as ~130 fs. Positron to electron (e+/e−) ratios >15% were observed for many thick disk and rod targets, with the highest e+/e− ratio reaching ~50% for a Pt rod. The inferred pair yield was ~ few ×10^10^ with emerging pair density reaching ~10^15^/cm^3^ so that the pair skin depth becomes < pair jet transverse size. These results represent major milestones towards the goal of creating a significant quantity of dense pair-dominated plasmas with e+/e− approaching 100% and pair skin depth ≪ pair plasma size, which will have wide-ranging applications to astrophysics and fundamental physics.

If a dense e+e− pair-dominated plasma can be created in the laboratory with sufficient volume, it has far reaching applications to astrophysics, fundamental physics and innovative technologies^1^. Pair creation using ultra-intense short-pulse laser irradiating high-Z solid targets has many advantages over accelerator-based pair creation, including higher pair density and higher yield. Previous experiments using lasers of intensity ~10^19^–10^20^ W.cm^−2^ irratiating gold disks have successfully demonstrated pair creation. However, the reported e+/e− ratios were ≤ few percent and the inferred pair density (~10^13^/cm^3^)^2^,^3^,^4^ was too low to satisfy the condition: pair skin depth < pair plasma size, the conventional definition of a “plasma”. Here we report new pair creation experiments using ~100 Joule pulses of the Texas Petawatt Laser^5^ to irradiate gold and platinum disk and rod targets, which produced much higher e+/e− ratio and higher pair density than previous laser-solid experiments. The ultimate goal of laser-solid pair creation is to produce a sufficiently large quantity of dense pair-dominated plasma with the highest yield^1^,^11^, so that the properties and behaviors of such plasmas can be studied in detail.

There are two different approaches to create pairs with lasers. The first approach is to create pairs by directly irradiating high-Z solid targets with ultraintense lasers (the “1-step” process). Lasers with intensity >1.4 × 10^18 ^W.cm^−2^ irradiating solid targets couple 10–50% of their energy to hot electrons^6^,^7^ with effective temperature kT > mc^2^ 8. When electrons with energy exceeding 1.02 MeV impact high-Z target ions, pairs are created via the Trident and Bethe-Heitler (BH) processes^9^,^10^,^11^,^12^,^13^,^14^,^15^. Cowan *et al.*^16^,^17^ first demonstrated “1-step” pair creation using the Nova petawatt (PW)-laser irradiating solid gold targets. This was followed by by Chen *et al.*^2^,^3^,^4^ using the Titan and Omega-EP lasers to irradiate ~mm thick gold disks with intensities up to ~10^20^ W.cm^−2^. The emergent e+/e− ratio reached a few percent and the positron yield reached ~10^11^ per kJ of laser energy with inferred pair density ~10^13^/cm^3^ 2,3,4. The second approach is to first create a relativistic electron beam via LWFA^18^ by irradiating an underdense gas jet, and then injecting the electron beam into a high-Z converter to create pairs (the “2-step” process). Gahn *et al.*^19^ first demonstrated the “2-step” process using table-top high-rep-rate lasers. Recently, using the ASTRA-GEMINI laser at RAL, Sarri *et al.*^20^ demonstrated the generation of quasi-neutral narrow 200–600 MeV e+e− beams using the “2-step” process. Due to the fewer hot electrons accelerated by LWFA in a gas jet (~10^9^ )^20^ than in laser-solid interactions (~10^12^–10^13^)^6^,^7^, the total number of pairs created using the “2-step” process is lower than the “1-step” process, but the emergent e+e− beam can reach higher energy (>100 MeV), lower divergence and higher density^20^,^21^,^22^. Hence the applications of laser pair creation using solid targets (1-step) versus gas jets (2-step) are different and complementary (see **Discussion**). Here we focus on the “1-step” approach as we are more interested in high yield applications. For a given laser energy irradiating solid targets, higher intensity is expected to create more energetic pairs which escape more easily from a thicker target, while a shorter pulse can create pairs at higher density and produce stronger internal and sheath fields to assist the positron escape. This motivates the pursuit of pair creation using more intense laser with shorter pulse to irradiate thicker targets.

The Texas Petawatt laser (TPW) in Austin, Texas^5^ was upgraded in 2012 with a new f/3 dielectric off-axis parabolic mirror donated by Los Alamos National Laboratory, allowing it to focus >100 J of energy from pulses as short as 130 fs to peak intensities >10^21 ^W.cm^−2^. We performed ~130 shots on Au, Pt targets. 15% of shots reached peak intensities ≥10^21^ W.cm^−2^. Hot electrons were heated to kT ≥ 15 MeV, and copious gamma-rays and pairs were observed. Our most important new findings include: (1) The observed e+/e− ratio exceeds 15% in 20 shots using thick disk and rod targets, reaching ~50%+/−10% for one Pt rod. (2) We infer a maximum emerging pair density ~10^15^/cm^3^, so that the effective pair skin depth = (mc^2^/8πe^2^n_+_)^1/2^ becomes smaller than the plasma size. (3) Long narrow rod targets produce higher observed e+/e− ratios than disk targets. (4) For thick disk and rod targets, Pt produces higher observed e+/e− ratio than Au. Hence Pt rod may be the favorite target for creating pair-dominated plasmas^1^,^11^ in future laser-solid experiments.

## Results

**Figure 1:**
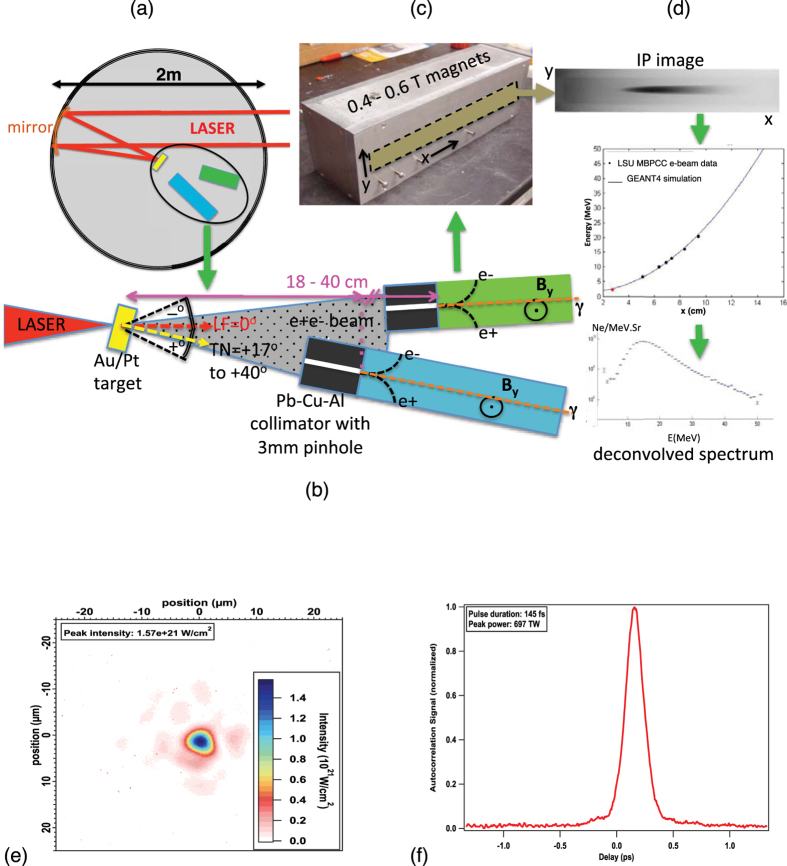
Experimental Setup. (**a**) Sketch showing the top view of the TPW target chamber with placement of the laser target at chamber center and two magnetic spectrometers viewing the target back. In some shots a third spectrometer views the target front side. (**b**) Sketch showing the laser target orientation, magnetic spectrometers and collimators for a typical disk target experiment. All angles are meansured from the laser forward (LF = 0^o^) direction and all distances are measured from the target front center. Positive angles sweep clockwise from LF and negative angles sweep counter-clockwise from LF. (**c**) Picture of a magnetic spectrometer showing the location of the 2.4 cm-wide imaging plate (IP) slot. (**d**) Flow chart illustrating the conversion of an electron IP image into an energy spectrum. Middle picture shows the spectrometer response curve. (**e**) Sample laser focal spot intensity distribution, with the peak intensity displayed in the upper left corner. (**f**) Sample laser pulse time profile with the pulse width displayed in the upper left corner.

**Figure 2:**
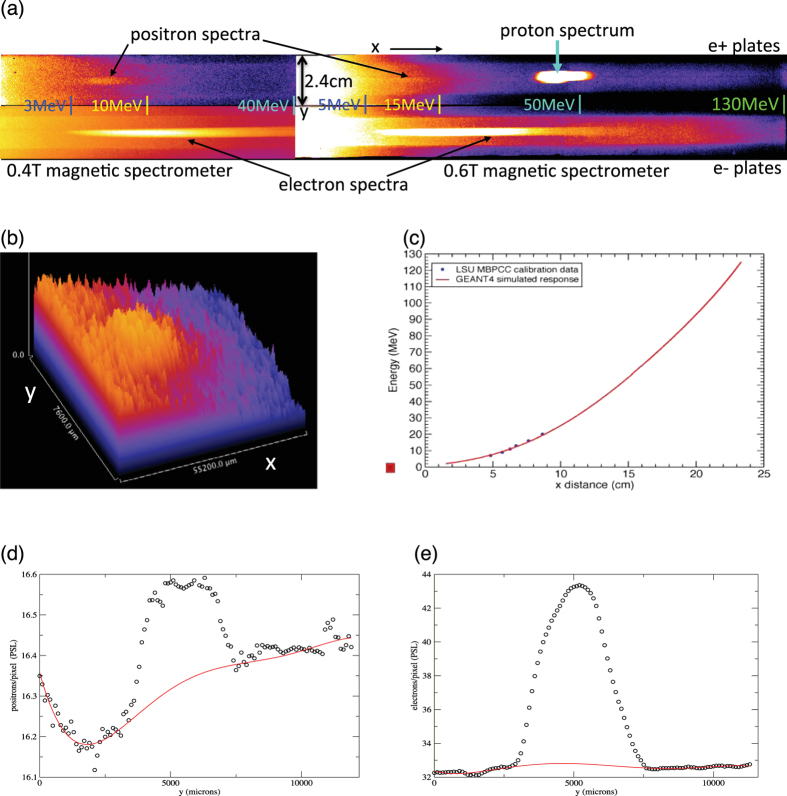
Image plate signals. (**a**) Sample IP images showing the e+ (top), e− (bottom) and proton signals in PSL units from two different magnetic spectrometers. Left images are from the low-energy 0.4T magnetic spectrometer for a 1 mm Pt target (Shot 6701). Right images are from the high-energy 0.6T magnetic spectrometer for a 1 mm Au target (Shot 3789). Markers denote approximate energy scales along the x-axis. (**b**) 3D contour map of the positron signal for Shot 6701 (top left IP of Fig. 2(a)) showing the nonuniformity of the background relative to the positron signal strength and pixel-level noise. (**c**) Electron energy versus distance along x for the 0.6T spectrometer generated using GEANT4 simulations (red curve) and calibrated against clinical e-beam data (blue dots). The agreement is better than 0.5 MeV from 6 MeV to 20 MeV. (**d**) Energy-integrated positron PSL profile (black dots) across the magnet gap (y-axis) for Shot 6701. Positron signal shows up as a ~4 mm wide “bump” centered around the middle of the gap. Red curve denotes best polynomial fit to the background. (**e**) Energy-integrated electron PSL profile (black dots) across magnet gap for Shot 6701. Electron signal forms a ~5 mm wide “bump” centered around the middle of the gap. Red curve denotes best polynomial fit to the background.

The experiments were carried out in the 2m-diameter solid-target chamber TC1 of TPW with heavy radiation shielding. Figure 1 shows schematically the experimental setup, sample laser focal spot size and pulse profile. Laser and target parameters for all shots are summarized in Table 1. Charged particle signals are recorded on Fuji imaging plates (IP) attached onto NdFeB magnetic spectrometers (Fig. 1c, see **Methods**). Figure 2a shows sample IP images of electron, positron and proton (e+e − p) spectra after conversion to PSL units^23^,^24^. The positron signals are clearly visible in both the low-energy (0.5–45 MeV) and high-energy (1.5–130 MeV) IP images. Proton signals from target surface contaminants are also seen in many shots, with energies ~1–2 MeV. Figure 2b highlights the positron signal compared to the background level (mostly secondary x-rays produced inside the spectrometer), which is highly nonuniform along and across the magnet gap. Background subtraction is performed using detailed polynomial fits (Fig. 2d,e), and the procedure is certified using shots with no positrons (Al targets and e-beams, see **Methods**). The spectrometer response curves (Figs 1d and 2c) are generated using GEANT4 simulations^25^ based on detailed magnetic field measurements, and then calibrated using clinical e-beams of known energies at the LSU Mary Bird Perkins Cancer Center (MBPCC) at Baton Rouge, Louisana^26^.

**Figure 3:**
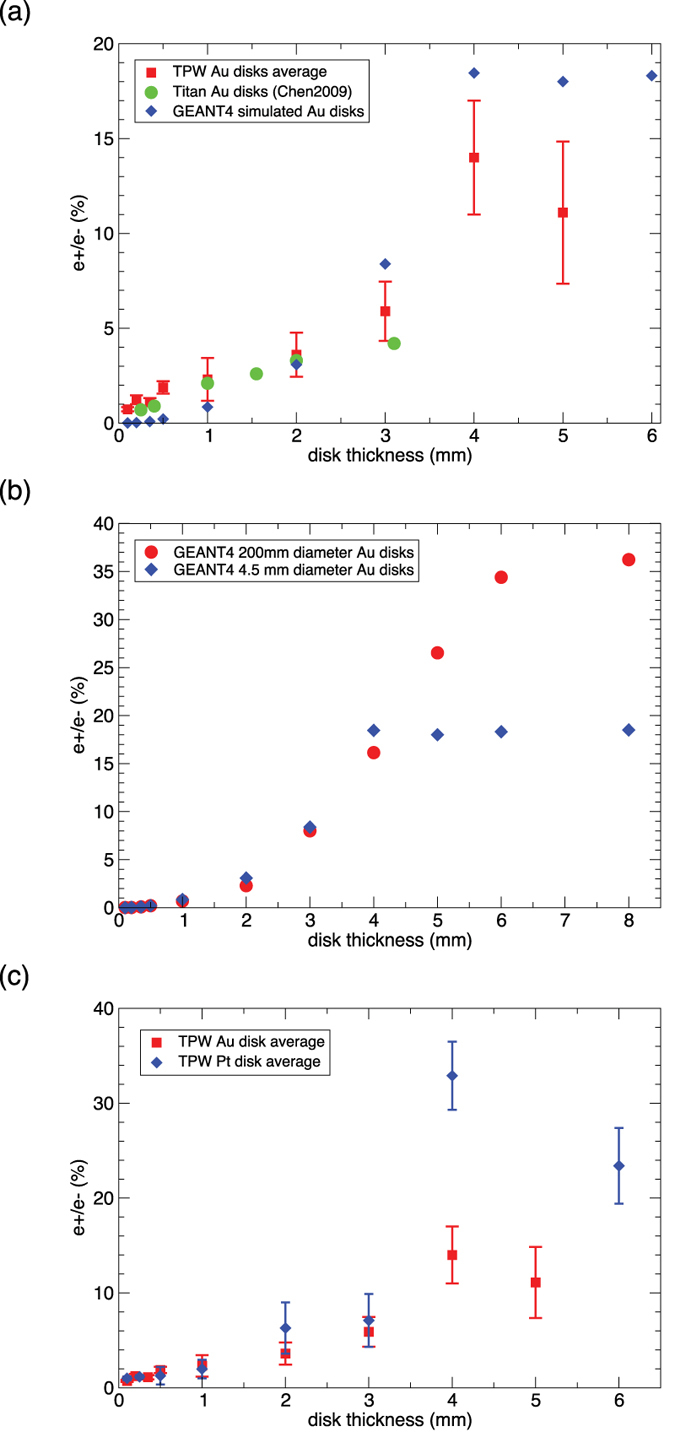
Positron/electron ratio for disk targets. (**a**) Observed TPW e+/e− ratio versus target thickness for Au disks with diameter up to 4.5 mm (red square), compared to Titan Au disk data (green dot^2^,) and GEANT4 simulations (blue diamond^25^). TPW and Titan data agree with each other below 3 mm thickness, but the TPW data rises steeply from 3 mm to 4 mm thickness, clearly deviating from the linear trend of Titan data. Each data point is the average of multiple shots and detectors. The number of data points (shots x detectors) used in the average are: 0.1 mm (2), 0.2 mm (3), 0.35 mm (3), 0.5 mm (12), 1 mm (19), 2 mm (11), 3 mm (12), 4 mm (10), 5 mm (2). Error bars include the spread among different data points and the intrinsic uncertainty of each data point. (**b**) Comparison of GEANT4 simulated e+/e− ratios for Au disks of 4.5 mm diameter (blue diamond) to disks of 200 mm diameter (red dot). This confirms that the observed decrease above 4 mm in Fig. 3(a) is an artifact of the small disk diameter. If we had used much larger diameter disks, the e+/e− ratio should continue to rise beyond 4 mm thickness (red dots). (**c**) Comparison of Au disk e+/e− ratios with Pt disk ratios. For thickness ≥ 4 mm, the observed Pt e+/e− ratio is > twice the Au ratio. The number of data points (shots x detectors) used in the Pt averages are: 0.1 mm (2), 0.25 mm (2), 0.5 mm (3), 1 mm (6), 2 mm (2), 3 mm (2), 4 mm (2), 6 mm (2). Error bars include the spread among different data points and the intrinsic uncertainty of each data point.

Figure 3a compares the TPW e+/e− ratio vs. disk target thickness with published Titan Au data^2^ and with GEANT4 simulations^25^. For targets thinner than 3 mm, TPW and Titan data basically agree, but the TPW ratio rises steeply from 3 mm to 4 mm thickness and clearly deviates from the linear trend extrapolated from the Titan results, which had no published data above 3 mm. TPW intensity was higher than Titan and produced higher energy electrons, which in turn create higher energy bremsstrahlung photons and pairs that can escape more easily from thick targets, while the primary electrons are more attenuated by thicker targets. Our data agree qualitatively with the trend predicted by GEANT4 (Fig. 3a blue diamonds), which also shows that the decline above 4 mm is due to the small disk diameter (4.5 mm) used in our experiment. Figure 3b shows that, if we had used much bigger diameter disks (»4.5 mm), the e+/e− ratio should continue to rise beyond 4 mm thickness (red dots). Quantitatively, GEANT4 underpredicts the e+/e− ratio for thicknesses ≤1 mm, and overpredicts the ratio for thickness ≥3 mm (Fig. 3a). The underprediction for thin targets is likely caused by GEANT4 not including the Trident process^10^ or sheath electric fields^27^, both of which should increase the e+ yield for thin targets. This may also partially explain why the Titan data trend appears linear. However the GEANT4 overprediction for thick targets (≥3 mm) remains to be understood since the Trident process and sheath field should play little role for thick targets. We note that the predicted absolute positron yield actually tops out at ~1–2 mm thickness^25^. Thus the monotonic rise of the emergent e+/e− ratio with thickness (Fig. 3b red dots) is mainly caused by the increasing absorption of primary electrons with increasing thickness. Figure 3c compares the e+/e− ratio of Au versus Pt disk targets. We see that the observed e+/e− ratio for Pt jumps to more than twice that of Au for thicknesses ≥4 mm. This is caused by the reduction of hot electrons emerging from thick Pt targets, while the absolute positron yield stays roughly the same for Au and Pt. Pt has five times the electrical resistivity of Au, which likely reduces the return current of ambient electrons in the target and inhibits the propagation of the hot electrons^7^.

**Figure 4:**
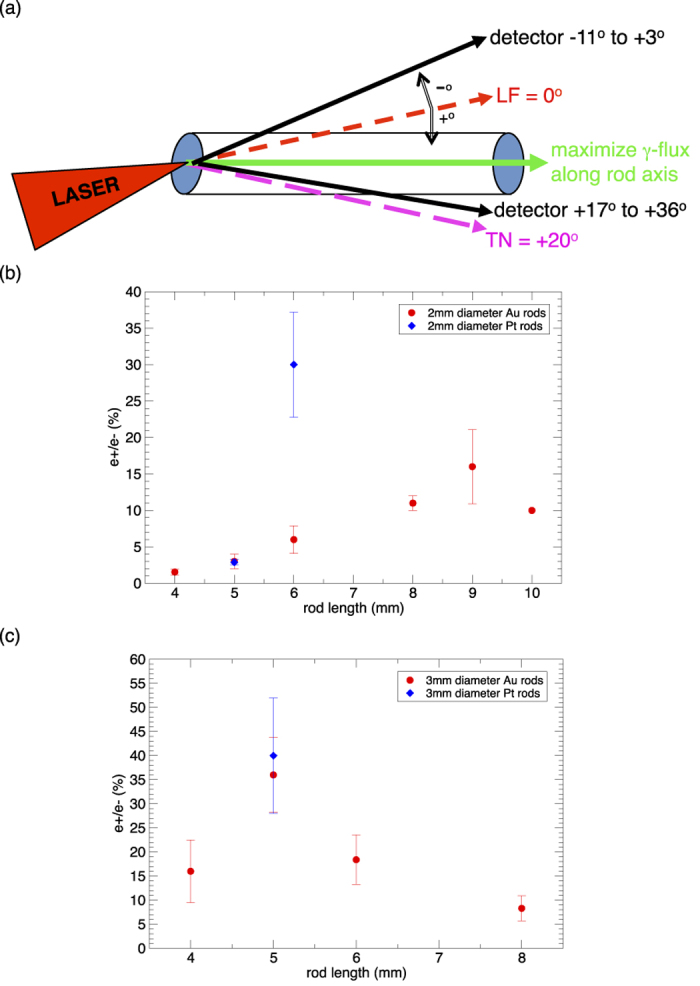
Positron/electron ratio for rod targets. (**a**) Sketch showing long narrow rod target experimental setup, which maximizes hot electron and gamma-ray absorption along the rod axis, and minimizes absorption of pairs emitted sideways. The detectors are positioned to see emissions from the side of the rod instead of the back of the rod. The rod axis typically lies mid-way between LF and TN. (**b**) Plot of the highest e+/e− ratio for 2 mm-diameter Au and Pt rods versus rod length, showing that Au rods peak at 9mm length and that Pt rods can reach higher ratios than Au rods. Here we choose only the highest ratio among the different detectors for each shot, and then average this ratio for all rods of the same length. The number of Au data points used in each average: 4 mm (2), 5 mm (3), 6 mm (3), 8 mm (2), 9 mm (2), 10 mm (1). The number of Pt data points used in each average: 5 mm (2), 6 mm (2). Error bars represent only data spread among the different shots. There is only one 10 mm long Au rod, hence there is no spread. The intrinsic uncertainty of each shot (not shown) ranges from 10 to 20 percent of the measured e+/e− ratio. (**c**) Plot of the highest e+/e− ratio among different detectors for 3 mm-diameter Au and Pt rods versus rod length. The number of Au data points used in each average: 4 mm (2), 5 mm (3), 6 mm (2), 8 mm (4). The number of Pt data points used in the average: 5 mm (2). The two Pt rod values are 52%+/− 10% and 28%+/− 4%. Error bars represent data spread among the different shots. The intrinsic uncertainty of each shot (not shown) ranges from 10 to 20 percent of the measured e+/e− ratio.

Our most important result comes from the rod targets (Fig. 4). The idea is to irradiate the end of a long narrow rod so that the primary hot electrons and their bremsstrahlung photons propagate mainly along the rod axis (Fig. 3a). Away from the rod axis, we should detect a higher e+/e− ratio by avoiding most of the primary electrons. Moreover, a long narrow rod provides more optical depth for the bremsstrahlung emission and pair production along the rod axis, while it minimizes the absorption of pairs emitted sideways. This idea is largely confirmed by our rod target data: most of our rod targets produce maximum e+/e− ratios >10% when observed at angles away from the rod axis towards target normal (TN) direction (Fig. 4b,c). Shots using 3 mm diameter rods (Fig. 4c) produced higher e+/e− ratios than 2 mm diameter rods (Fig. 4b), raising the hope that using rods with diameter >3 mm may produce even higher ratios. Again Pt rods work better than Au rods, with the e+/e− ratio of one 3 mm diameter Pt rod reaching 52%+/−10%. These results suggest that Pt rods may be the preferred target to create pair-dominated plasmas with e+/e− >50% at birth, independent of any energy selection or magnetic focussing schemes to further increase the e+/e− ratio downstream^28^.

The highest inferred emerging pair density comes from our 0.35 mm thick Au disks. For these targets the observed positron yields were ~3 × 10^10^/str. Integrating over an emission cone of 25^o^ (~laser incident angle, see also^3^,^25^,^29^), we conservatively estimate a total positron yield of N_+_~1.8 × 10^10^ for 100 J of laser energy. Detailed GEANT4 simulations^25^ show that in this case the emerging positrons are concentrated in a pill box of diameter D ~ 0.4 mm and thickness cΔt ~ 90 μm, where Δt =3 00 fs is the pulse duration of the emerging positrons. Hence the inferred positron density in this case is n_+ _~ 1.8 × 10^10^/π(0.02)^2^(0.009) = 1.6 × 10^15 ^cm^−3^. At this density the “pair skin depth” c/ω_pair _= c/(8πn_+_e^2^/m)^1/2^ is ~ 0.1 mm. Hence Dω_pair_/c ~ 4, qualifying the pair jet as a “pair plasma” using a common definition of “plasma^30^” (see **Discussion**). However, for many relativistic kinetic processes the more relevant length scale to compare with D is the “relativistic pair skin depth” = cγ^1/2^/ω_pair_ where γ is the average Lorentz factor of the pairs^20^ (see **Discussion**). In this case our 1 mm thick Au target data actually lead to a smaller “relativistic pair skin depth” relative to the plasma size D, because their positron Lorentz factor is much lower than those for 0.35 mm thick targets (see below). The average positron Lorentz factor of our 1 mm thick Au targets with the highest e+ yield (N_+ _~ 2 × 10^10^) was γ ~ 14. Hence γ^1/2 ^= 3.7, and the ratio Dω_pair_/cγ^1/2 ^= 1.1, marginally >1. As Sarri *et al.*^20^ point out, this ratio is independent of D, and scales as (N_+_/γΔt)^1/2^. For laser-solid interactions, N_+_ scales with laser energy^15^. Hence our results demonstrate that future ultraintense lasers with Δt < 100 fs and energy ≫ 100 J irradiating mm-thick Au or Pt targets should easily create a pair plasma with Dω_pair_/cγ^1/2^ ≫ 1.

**Figure 5:**
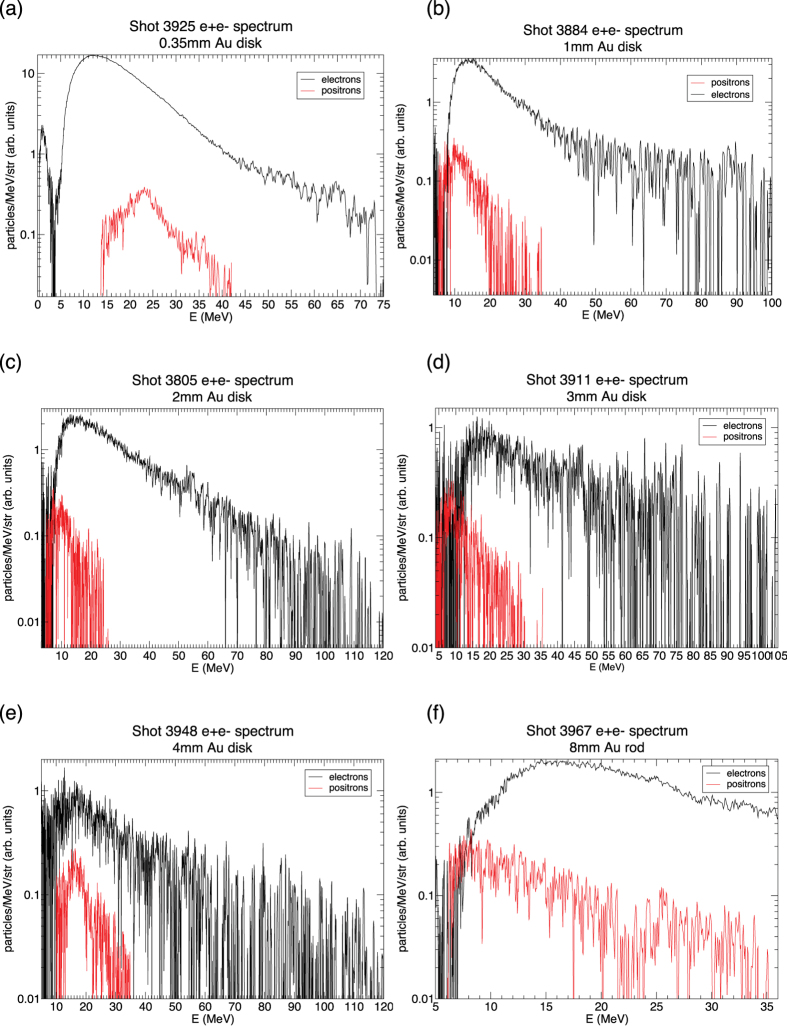
Positron and electron spectra. Deconvolved electron and positron spectra for six Au targets showing the variation with thickness (**a–e**) and geometry (**f**). All spectra are recorded by detectors near laser forward (LF) direction (+3^o^ to −10^o^). All electron spectra peak at ~10–16 MeV while the positron peak ranges from ~6 MeV to 23 MeV. The positron high energy slope is softer than the electron slope for all disk targets (**a–e**), but is almost as hard as the electron slope for the rod target (**f**). All electron spectra show a deficit of low energy electrons. Some electron spectra show a second harder component beyond ~ 40 MeV (**a,b**). The deep troughs seen in some spectra are due to IP or scanner defects.

Figure 5 shows sample deconvolved positron and electron spectra for Au targets. While the positron peak energy varies widely (6–23 MeV), the electron peak energy is remarkably stable (10–16 MeV) independent of target geometry, thickness and material. Positron kT is typically ~1/2 of the electron kT except for the rod target, for which the positron slope is almost as hard as the electron slope. This is likely due to the convolution of positrons emitted by different parts of the rod. All of our electron spectra show a strong deficit of electrons below a few MeV. This spectral behavior differs from those reported for other PW laser experiments^3^,^7^,^16^,^31^, and suggests that TPW electrons below a few MeV are more strongly attenuated and/or refluxed back into the target. This new regime of hot electron transport requires further investigation.

**Figure 6:**
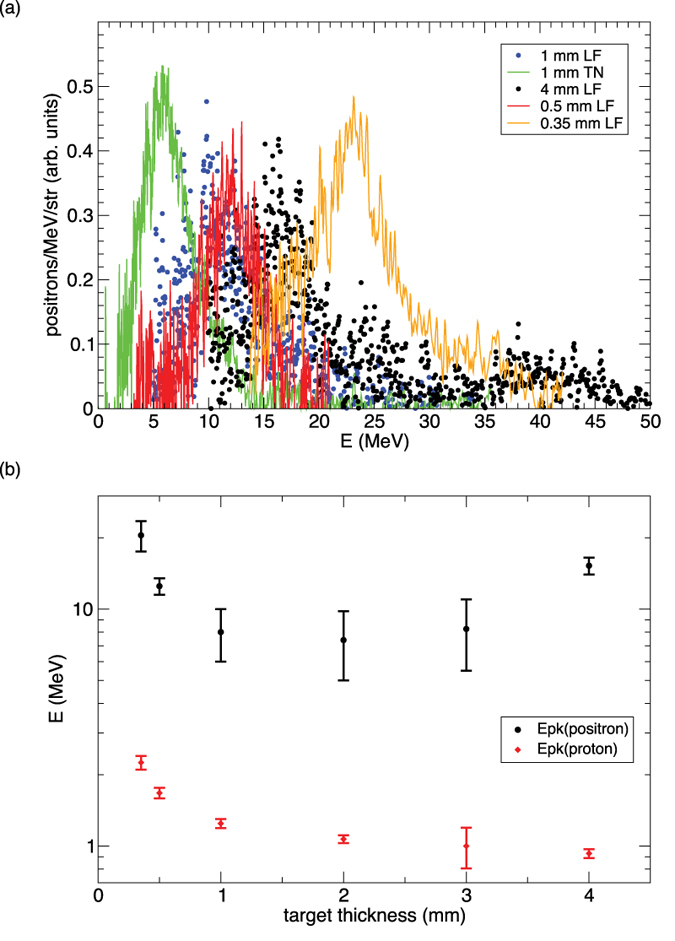
Positron and proton peak energies. (**a**) Comparison of positron spectra for Au disks of different thicknesses and at different detector angles. Here LF refers to −8^o^ and TN refers to +36^o^. Amplitudes have been renormalized to show all spectra on the same scale. (**b**) Thickness dependence of positron peak energy (black dot) vs. proton peak energy (red diamond) for Au disks. The positron peak energy reaches a minimum at ~2 mm thickness, while the proton peak energy decreases monotonically with increasing thickness. Each upper error bar corresponds to the highest peak energy measured near LF (−5^o^ to −9^o^), and each lower error bar corresponds to the lowest peak energy measured near TN (+36^o^ to +40^o^). Peak energy at LF is always higher than at TN (see Fig. 6(a)).

Figure 6a compares the positron spectra of various target thickness and detector angle for Au disks. The positron peak energy ranges from ~6 MeV up to 23 MeV. Figure 6b compares the thickness dependence of the positron peak energy E_+_ and proton peak energy E_p_. While E_p_ decreases monotonically with thickness, E_+_ has a minimum around 2 mm. The decrease of E_p_ and E_+_ (below 2 mm) with increasing target thickness is consistent with sheath field acceleration^27^,^32^ since the sheath field is stronger for thinner targets. However, the reversal of E_+_ from 2 mm to 4 mm is likely caused by increasing attenuation of low-energy positrons by the thicker targets. Since E_p_ is much smaller than E_+_, the protons must experience only a small fraction of the sheath potential seen by the positrons. To help understand these results we have performed particle-in-cell (PIC) simulations^30^ to explore the plasma physics of positron and proton acceleration.

**Figure 7:**
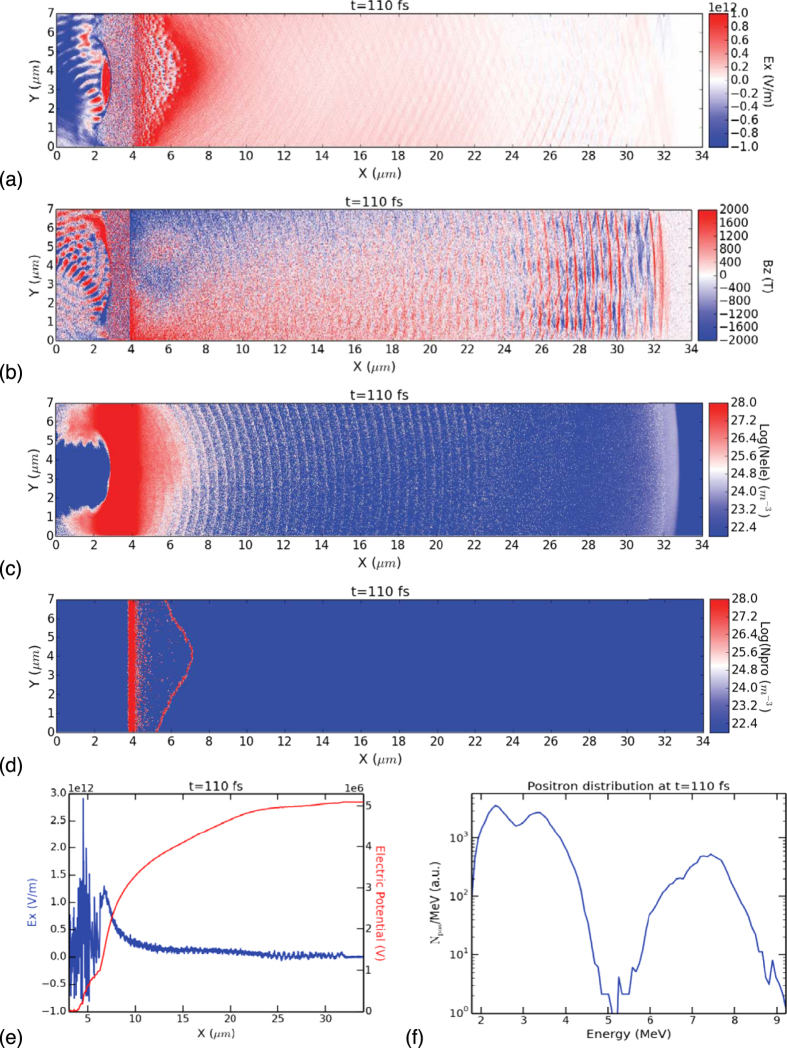
PIC simulation of TPW irradiating Au target. We show spatial profiles of (**a**) electric field E_x_, (**b**) magnetic field B_z_, (**c**) electron density N_e_, (**d**) proton density N_p_, at 110 fs. (**e**) Lineout of sheath electric field E_x_ (blue curve) and sheath potential (red curve) along y =3.5 μm. The total sheath potential reaches ~5 MeV in this run. (**f**) Positron energy distribution near the upper right boundary at 110 fs shows two distinct peaks at ~2.5 MeV and ~7.5 MeV respectively, separated by the 5 MeV sheath potential. At this time the protons have travelled only 1–3 μm (Fig. 7d) and gained ~1 MeV of sheath potential (Fig. 7e).

Figure 7 shows the results of a 2-dimensional PIC simulation using the EPOCH code (see **Methods**). A 1 μm wavelength laser with intensity = 10^2^ ^1^W.cm^−2^, pulse duration = 160 fs and Gaussian focal spot diameter = 2 μm irradiates from left a “solid gold” plasma (electron density = 4200 × critical density and ion mass = 197 × proton mass), at an incident angle of 15^o^ and electric field parallel to target surface (s-polarized). The simulation box has physical dimensions of 34 μm × 7 μm, with cell size = c/ω_e _= 2.5 × 10^−3 ^μm (see **Methods** for details). The Au target has thickness of 1 μm, located between ×= 3 μm and 4 μm. An exponential density ramp is provided in front of the target to simulate the preplasma (TPW laser contrast ~ 10^−7^ 5). Even though such a thin target is unrealistic compared to the mm thick targets of our experiment, it should allow the hot electrons accelerated at the target front to penetrate the target, exit the back surface, and propagate for sufficient distance (30 μm) to create a meaningful sheath potential. Passive tracer particles are used to model the positrons (initialized throughout the target) and protons (initialized only at the target back surface). The idea is to model some aspects of the sheath acceleration of positron and proton from first principles. Figure 7a–d show spatial profiles of E_x_, B_z_, N_e_ (electron density) and N_p_ (proton density) at 110 fs, just before the hot electrons reach the right boundary. Figure 7e shows the lineout at y=3.5 μm of E_x_ and the sheath potential (S E_x_dx) which reaches ~5 MeV at this stage. Figure 7f shows the energy distribution of positrons reaching the upper right boundary near the laser forward direction. The positrons form two distinct peaks separated by ~5 MeV. The low energy peak corresponds to positrons directly accelerated by the laser prior to the formation of the sheath potential, which cannot occur in a real experiment since the pairs are created deep inside the thick target and cannot experience any laser acceleration. The high energy peak at ~7.5 MeV corresponds to positrons accelerated by both the laser and the sheath field. Even though real positrons created inside our thick targets are not subject to direct laser acceleration, it turns out they are in fact born with energies peaking at ~1.5–2 MeV^25^ due to the convolution of the pair production cross section^10^ with the TPW bremsstrahlung spectrum^29^. Hence the prediction of the ~7.5 MeV high energy peak in Fig. 7f is semi-realistic. In the real experiment, space is 3D and a fraction of the hot electrons is attenuated by the mm thick target, both of which reduce the sheath potential. On the other hand, the focal spot size (Fig. 1e) and target y-dimension are larger, both of which increase the sheath potential. Hence it is satisfying that the prediction of Fig. 7f lies within the range of the observed positron energies (Fig. 6). At the same time, Fig. 7d shows that the protons have travelled only 1–3 μm from the target at 110 fs. From the red curve of Fig. 7e this distance translates into a sheath potential of ~1 MeV, thus qualitatively explaining the large difference between the proton and positron energies (Fig. 6b). The PIC simulation also shows that positrons along the target normal direction reach lower energies than those along the laser forward direction, in agreement with observations (Fig. 6), since more electrons are emitted towards laser forward than target normal. In summary, even though our PIC simulation can only model a very thin Au target in a small 2D box, it seems to capture some of the essential physics of positron and proton acceleration, and qualitatively explain the observed positron and proton energies from first principles.

## Discussion

The most important results of our experiments are: (a) Pt targets can lead to higher emergent e+/e− ratio than Au targets, and (b) long narrow rods allow pairs to escape off-axis with higher e+/e− ratio than disks. Since one of our 3 mm diameter Pt rods produced the highest e+/e− ratio observed so far (52%+/−10%) for laser-solid experiments, we will explore using even bigger Pt rods to reach higher e+/e− ratios. We infer a “pair skin depth” ~4 times smaller than the transverse pair jet size, and a “relativistic pair skin depth” marginally smaller than the transverse pair jet size^20^. Since our pairs are imbedded in a nonneutral electron plasma of higher density, it is debatable what the best definition of a “pair plasma” should be. Different plasma instabilities also have different dependences on the Lorentz factor^7^,^30^. Hence it may be too simplistic to use a single kinetic length scale to characterize a relativistic “pair plasma”. Despite this, there is little doubt that future laser pulses with intensity >10^21^ W.cm^−2^, pulse duration <100 fs and energy ≫100 J irradiating solid Au and Pt targets, should create more pairs with higher density that will easily satisfy any “pair plasma” definition. A sufficiently large quantity of dense pair-dominated plasma will have wide ranging applications to laboratory astrophysics (simulating pulsar winds and gamma-ray bursts) and fundamental physics (Bose-Einstein condensate of positronium^33^ and gamma-ray amplification via stimulated annihilation radiation or GRASAR^1^,^33^).

It is useful to highlight here the key differences and complementarity between the 1-step (laser-solid^2^,^3^,^4^) and 2-step (laser-gas jet-converter^19^,^20^) approaches to laser pair creation, since both are actively pursued. The 1-step approach creates ~ MeV pairs with a broad beam and hence lower pair density. The 2-step approach creates ≥0.1 GeV pairs with a narrow beam and hence higher pair density^20^. The 1-step approach produces higher pair yield (observed N_+_ ~ fewx10^10^–10^11^ 3), and N_+_ scales with laser energy. The 2-step approach produces lower yield (N_+_~fewx10^7^ observed and ~10^9^ simulated^20^), and it is unclear how to increase N_+_ with laser energy. The advantage of the 2-step approach is that the LWFA electron beam can readily reach GeV energies and their bremsstrahlung photons can penetrate cm-thick converters to produce quasi-neutral pairs with e+/e− ratio ~100%^20^, whereas the 1-step approach will have to wait for much higher laser intensities to reach GeV electron energies. But our rod targets may provide an alternative approach to achieving high e+/e− ratio. Interestingly, the results reported here and in Sarri *et al.*^20^ both give similar Dω_pair_/c<γ>^1/2^ values of ~1, despite their very different N_+_, Lorentz factors and pulse durations. Thus both approaches are at the “threshold” of achieving a “pair plasma” however it is defined, but of very different dimensions and properties. Looking ahead, we believe that the 1-step approach will be more useful for applications requiring large amount of pairs at low energies such as the creation of a BEC of positronium, since it is easier to slow MeV pairs than GeV pairs, while the 2-step approach will be more useful for applications requiring narrow e+e− beams at high energies, such as particle accelerators and advanced light sources. Both approaches can produce pair plasmas relevant to astrophysics^1^.

## Methods

### Magnetic Spectrometers

Three positron-electron-proton (e+e − p) spectrometers made with NdFeB magnets of 0.4T to 0.6T and 3 mm diameter pinholes were used to measure the e+e − p spectra at distances of 18–40 cm from the target (Fig. 1). The spectrometers cover the energy ranges 0.5–45 MeV, 1–60 MeV and 1.5–130 MeV respectively (Fig. 2). One spectrometer was positioned near the LF(=0^o^) direction behind the target (–11.5^o^ to +3^o^, positive angle is measured clockwise from LF, negative angle counter clockwise from LF, cf. Figure 1b), one positioned near the TN direction (+17^o^ to +40^o^) behind the target, plus one facing the target front side at various angles in some shots. The laser was s-polarized and the laser incident angle varied between 17^o^ and 45^o^ (see Fig. 1b). The spectrometers were calibrated using the LSU Mary Bird Perkins Cancer Center clinical e-beams of known energy^26^. Up to 10 cm of Pb-Cu-Al-plastic stack collimators with 3 mm pinholes were attached to the front of the spectrometers to provide shielding and collimation. Spectra were recorded using Fuji imaging plates (#BAS-IP-MS) and FLA7000 scanner^23^,^24^.

### Positron data

Even though the positron signal is weak compared to the internal x-ray background, it is concentrated in a ~4 mm wide strip along the center of the magnet gap (Fig. 2). Hence we developed a background subtraction procedure based on polynominal fits to the two dimensional background, using the optimization of R^2^ as a function of central pixels removed. This method produced robust background-subtracted signal for which the 1 − σ uncertainty is well-quantified. We tested this algorithm using Al target and clinical e-beam data, whose e+ IP backgrounds contain no real positrons, while their e- IP backgrounds are similar to those of Au and Pt shots. All such e+ IP images gave null (<1 − σ) positron signal after background subtraction. All Au and Pt data reported in this paper come from positron signals >3 − σ. To facilitate comparison of e+/e− ratios from all spectrometers, we include only positrons and electrons between 2 MeV and 50 MeV.

### GEANT4 simulations

We used GEANT4 to simulate bremsstrahlung and pair production by laser-driven hot electrons in Au and Pt targets. GEANT4 is a widely used object-oriented Monte Carlo code developed at CERN for nuclear and particle physics. We inject hot electrons starting with a trial spectrum, 160 fs pulse duration and beam opening angle of 15^o^ into the Au target, and then iterate the incident spectrum until the output spectrum agrees with the observed electron spectrum. The positron output from the final iteration is then collected at a hemispherical detector surrounding the target, as a function of energy, angle and time. To generate the magnetic spectrometer response curve E(x) (Fig. 2c) we input detailed 3-dimensional magnetic field data measured inside the gap and inject normal-incident electrons and positrons into the 3-mm diameter pinhole at 0.5 MeV energy intervals. The positions of electrons and positrons hitting the imaging plates are then recorded. The red curve of Fig. 2c represents the centroid position of the Monte Carlo electron distributions. The proton energy E_p_ can be determined using the positron E(x) curve by substituting the positron momentum with the proton momentum = (2E_p_m_p_)^1/2^ where m_p_ is the proton mass.

### PIC simulation

We carried out two-dimensional particle-in-cell (PIC) simulation using the EPOCH code which has been widely used to model high energy density physics and laser target interactions. The core algorithm of this code is the same as the PSC code^34^. Our simulation domain spans 34 microns in x and 7 microns in y. A solid-density gold plasma of kT = 2.5 keV and 1 micron thickness is located between x = 3 micron and x = 4 micron. A laser beam comes in from the lower left boundary, and hits the center of the target at 15^o^ from target normal. The laser has a peak intensity of 10^21^ W.cm^−2^, duration of 160 fs, wavelength of 1 micron and focal spot diameter of 2 microns. Behind the target (x > 4 micron) is 30 microns of vacuum. In front of the target (x < 3 micron) is a gold plasma whose density falls off exponentially (e-folding distance = 0.12 microns) from the target surface to model the preplasma created by the laser prepulse. We included two passive tracer particle species in the simulation. The first one represents positrons, which follow the initial electron distribution. The second one represents protons which are located in a thin layer at the target back surface. We used cell size equal to electron skin depth so that the grid measures (13804 × 2842), with 20 particles per cell in the target region. The time step is half of the inverse electron plasma frequency in the target. We ran the simulation up to t = 200 fs.

## Additional Information

**How to cite this article**: Liang, E. *et al.* High e+/e− Ratio Dense Pair Creation with 10^21^W.cm^−2^ Laser Irradiating Solid Targets. *Sci. Rep.* **5**, 13968; doi: 10.1038/srep13968 (2015).

## Figures and Tables

**Table 1:** Laser and Target Parameters.

**Laser Parameters**	**Range**	**Average**
Energy E	81–130 J	<E> = 103 J
Pulse Duration ΔT	128–245 fs	<ΔT> = 166 fs
Peak Power P	450–802 TW	<P> = 640 TW
%Energy in 10 μm circle	40–80%	<%E> = 65%
Laser Incident angle θ	17o – 40o	<θ> = 25o
Peak Intensity on target I	3 × 10^20^–1.9 × 10^21 ^W.cm^−2^	<I> = 7 × 10^20^ W.cm^−2^
	(15% of shots reached I ≥ 1021 W.cm^-2^)	
Target Parameters		
Disks	Diameter	Thickness
73 gold	2 mm–4.5 mm	0.1 mm–5 mm
18 platinum	2 mm–4.5 mm	0.1 mm–6 mm
Rods	Diameter	Length
30 gold	2 mm–3 mm	4 mm–1 cm
9 platinum	2 mm–3 mm	4 mm–6 mm
